# Hematologic toxicities of chemotherapy in breast and ovarian cancer patients carrying *BRCA1/BRCA2* germline pathogenic variants. A single center experience and review of the literature

**DOI:** 10.1007/s10689-023-00331-6

**Published:** 2023-04-29

**Authors:** Ketty Hu-Heimgartner, Noémie Lang, Aurélie Ayme, Chang Ming, Jean‑Damien Combes, Victor N. Chappuis, Carla Vazquez, Alex Friedlaender, Aurélie Vuilleumier, Alexandre Bodmer, Valeria Viassolo, José L Sandoval, Pierre O. Chappuis, S. Intidhar Labidi-Galy

**Affiliations:** 1 Department of Oncology, Hôpitaux Universitaires de Genève, 4, Rue Gabrielle Perret-Gentil, Geneva, 1205 Switzerland; 2 Department of Diagnostics, Hôpitaux Universitaires de Genève, Geneva, Switzerland; 3 Department of Clinical Research, Faculty of Medicine, University of Basel, Basel, Switzerland; 4 Infections and Cancer Epidemiology Group, International Agency for Research on Cancer, Lyon, France; 5 Center of Translational Research in Onco-Hematology, Faculty of Medicine, University of Geneva, Swiss Cancer Center Leman, Genève, Switzerland

**Keywords:** Breast cancer, Ovarian cancer, BRCA mutation, Toxicity, Febrile neutropenia, Haploinsufficiency, Chemotherapy, Therapy myeloid neoplasm

## Abstract

BRCA1 and BRCA2 play a central role in DNA repair and their germline pathogenic variants (g*BRCA*) confer a high risk for developing breast and ovarian cancer. Standard chemotherapy regimens for these cancers include DNA-damaging agents. We hypothesized that *gBRCA* carriers might be at higher risk of developing chemotherapy-related hematologic toxicity and therapy-related myeloid neoplasms (t-MN). We conducted a retrospective study of women newly diagnosed with invasive breast or ovarian cancer who were screened for *gBRCA1/gBRCA2* at Geneva University Hospitals. All patients were treated with (neo-)adjuvant chemotherapy. We evaluated acute hematologic toxicities by analyzing the occurrence of febrile neutropenia and severe neutropenia (grade 4) at day 7–14 of the first cycle of chemotherapy and G-CSF use during the entire chemotherapy regimen. Characteristics of t-MN were collected. We reviewed medical records from 447 patients: 58 g*BRCA1* and 40 gBRCA2 carriers and 349 non-carriers. *gBRCA1* carriers were at higher risk of developing severe neutropenia (32% vs. 14.5%, *p* = 0.007; OR = 3.3, 95% CI [1.6-7], *p* = 0.001) and of requiring G-CSF for secondary prophylaxis (58.3% vs. 38.2%, *p* = 0.011; OR = 2.5, 95% CI [1.4–4.8], *p* = 0.004). *gBRCA2* carriers did not show increased acute hematologic toxicities. t-MN were observed in 2 patients (1 *gBRCA1* and one non-carrier). Our results suggested an increased acute hematologic toxicity upon exposure to chemotherapy for breast and ovarian cancer among *gBRCA1* but not *gBRCA2* carriers. A deeper characterization of t-MN is warranted with the recent development of PARP inhibitors in frontline therapy in *gBRCA* breast and ovarian cancer.

**Supplementary Information:**

The online version contains supplementary material available at 10.1007/s10689-023-00331-6.

## Introduction

*BRCA1* and *BRCA2* are tumor suppressor genes playing a central role in the repair of DNA double-strand breaks through homologous recombination, a fundamental DNA repair process that maintains genome integrity during cell proliferation [1]. Carrying germline pathogenic variants in *BRCA1* or *BRCA2* (g*BRCA*) confer a high risk for developing breast or ovarian cancer throughout a patient’s life [2, 3]. The average cumulative breast cancer and ovarian cancer risks by age 70 in g*BRCA1* carriers are estimated at 72% and 44%, respectively, and for g*BRCA2* carriers, at 69% and 17% [3]. The reason why breast and ovary are the mainly affected organs by the increased risk of cancer remains unanswered. One explanation is hormonally driven: oxidative DNA damage occurring during each menstrual cycle needs efficient homologous recombination pathway repair and could be exacerbated in haploinsufficient BRCA1 cells [4–6].

Severe neutropenia and hematologic toxicities usually arise due to myelosuppressive chemotherapy [7]. Febrile neutropenia is defined as an absolute neutrophil count (or expected to fall below) < 0.5 × 10^9^/L with a single temperature > 38.3 °C or a sustained temperature > 38.0 °C for more than one hour [8]. It confers 15% higher risk of mortality than in patients without febrile neutropenia [9, 10]. Primary prophylaxis for the prevention of febrile neutropenia is based on the expected risk of febrile neutropenia with the planned chemotherapy regimen, adjusted with age, comorbidities or any other factors increasing the risk of febrile neutropenia. Primary prophylaxis is not recommended if the overall risk of febrile neutropenia is estimated to be less than 20% [10].

All the cells of carriers of *BRCA1* or *BRCA2* germline pathogenic variants are haploinsufficient for the gene product involved (alteration of a single allele). In these patients, carcinogenesis implies a somatic loss of the second allele either by loss of heterozygosity or somatic alteration of the second allele [1, 11, 12]. Preclinical data support the hypothesis that non-tumoral cells, through haploinsufficiency, present genomic instability and are more sensitive to DNA-damaging agents [11, 13–16]. There are conflicting data on whether germline *BRCA1/BRCA2* variants are associated with an increased incidence of developing febrile neutropenia. We previously reported that breast cancer patients with *gBRCA1* have a higher incidence of febrile neutropenia and grade 4 neutropenia under chemotherapy [17]. Post-hoc subgroup analyses on randomized trials suggested that breast cancer patients carrying g*BRCA* [18], but not ovarian cancer patients [19, 20], showed a higher incidence of acute hematologic toxicities under taxanes. Long-term follow-up of *gBRCA* carriers treated with poly-(ADP-ribose) polymerase (PARP) inhibitors suggested an increased incidence of therapy-related myeloid neoplasms, i.e. myelodysplastic syndrome and acute myeloid leukemia in *gBRCA* carriers [21–24]. Moreover, these patients are also at a higher risk of developing anthracyclines-induced cardiotoxicity [25].

We hypothesized that *gBRCA1/BRCA2* carriers developing breast or ovarian cancer might be at higher risk for developing chemotherapy-related acute hematologic toxicity and therapy-related myeloid neoplasms. If shown, such association could impact breast and ovarian cancer management among this particular subpopulation of patients.

## Material and methods

### Study design

We conducted a retrospective study of all women newly diagnosed with breast or ovarian cancer who underwent germline *BRCA1/BRCA2* testing between December 1995 and December 2018 at the Unit of Oncogenetics and Cancer Prevention, Hôpitaux Universitaires de Genève. The Geneva Ethics Committee approved the research protocol (CCER 15–158). Deceased patients were included without consent, and living patients were included after written informed consent was obtained.

### Inclusion and exclusion criteria

We identified eligible patients for our study from the database of the UOPC. Inclusion criteria were women newly diagnosed with breast and ovarian cancer who underwent *BRCA1/BRCA2* germline testing and received first line of neoadjuvant or adjuvant chemotherapy. Exclusion criteria were primary prophylaxis with G-CSF, metastatic breast cancer and the absence of available clinical data/follow-up.

### Data collection

All data were collected from medical records. Tumor characteristics and laboratory results were collected from pathology and laboratory reports. For ovarian cancer patients, we collected the following clinical data: age at diagnosis, type of chemotherapy regimen and timing (neoadjuvant or adjuvant), dates (beginning and end) of chemotherapy, number of cycles administered, tumor characteristics (FIGO stage, histotype and grade). For breast cancer patients, we collected the following clinical data: age at diagnosis, type of chemotherapy regimen and timing (neoadjuvant or adjuvant), dates (beginning and end) of chemotherapy, number of cycles administered, tumor characteristics (TNM stage, grade, estrogen/progesterone receptors and HER2 status).

### Hematologic toxicities

Regarding acute hematologic toxicities, we collected hematologic values (neutrophil count, leukocyte count, hemoglobin and platelets) at baseline, i.e. before the first cycle of chemotherapy (C1) and 7–14 days after its administration. Hematological toxicity was graded according to the *Common Terminology Criteria for Adverse Events* version 5.0 [8], with agranulocytosis defined as absolute neutrophil count < 0.5 × 10^9^/L. Febrile neutropenia was defined as absolute neutrophil count < 1 × 10^9^/L and fever > 38.3 °C. We reported whether the patients received G-CSF to complete the entire chemotherapy treatment, dose reductions of chemotherapy and the occurrence of febrile neutropenia. Long-term hematologic toxicity such as therapy-related myeloid neoplasms, i.e. myelodysplastic syndrome and acute myeloid leukemia were collected.

#### Endpoints

The primary endpoint was the incidence of febrile neutropenia at day 7–14 of the first cycle of chemotherapy. The secondary outcomes were the incidence of grade 3–4 neutropenia, G-CSF use and chemotherapy dose reduction during the entire chemotherapy regimen.

### Statistical analysis

Outcomes were compared in *gBRCA1*, *gBRCA2* and non-carriers. Absolute and relative frequencies were calculated for categorical data, whereas median, minimum and maximum values were determined for continuous data. Categorical data were compared using Fisher’s exact test. Continuous variables were compared using the Mann Whitney U test. Acute chemotherapy-related hematological toxicity frequencies were compared pair by pair by *BRCA1/BRCA2* status (g*BRCA1* vs. non-carriers; g*BRCA2* vs. non-carriers) and corresponding age-adjusted odds ratio with 95% confidence interval were calculated using multivariable logistic regression models. Details of missing data for each variable can be found in supplementary Table 1. A double-sided *p* value < 0.05 was considered significant. All analyses were performed with R software (version 4.1.0).

## Results

### Characteristics of the study cohort

We reviewed the medical records of 1078 patients, 472 of whom met the inclusion criteria of our study. Among them, 25 women were excluded from the analysis due to lack of information regarding clinical data (**supplementary Fig. **1). In total, 447 patients were included for analysis: 304 had breast cancer and 140 had ovarian cancer patients. Fifty-eight (13%) were identified with *gBRCA1*, 40 (9%) with *gBRCA2* and 349 (78%) were non-carriers. Among breast cancer patients, 32 (10%) were *gBRCA1*, and 26 (8.6%) were *gBRCA2* carriers. Among 140 ovarian cancer patients, 26 (18.6%) were *gBRCA1* carriers and 13 (9.3%) were g*BRCA2* carriers. No differences in age at diagnosis were observed according to *BRCA1/2* genotype, except for g*BRCA1* ovarian cancer patients being younger than non-carriers, as expected. Patients’ demographics, clinical and treatment characteristics are summarized in Table 1. Missing data are listed in **Supplementary Table 1**.

**Table 1:** Patients characteristics. Abbreviations: HGSOC, high grade serous ovarian cancer; TNBC, triple negative breast cancer

	Non-carriers	*BRCA1*	*p*	*BRCA2*	*p*
	349 (78%)	58 (13%)		40 (9%)	
**Age (median ; min-max)**	46.7 (16.7–83.6)	48.7 (24.2–70.5)	0.96	48.8 (30.8–74.2)	0.59
Breast	42.1 (16.7–78.2)	38.6 (24.2–68.1)	0.31	43.9 (30.8–61.9)	0.87
Ovarian	61.6 (26–83.6)	53.9 (40.5–70.5)	0.016	60.4 (45.2–74.2)	0.93
**Histology**					
**Breast**			< 0.0001		0.64
TNBC	63 (25.6)	22 (68.8)		8 (30.8)	
Other	183 (74.4)	10 (31.2)		18 (69.2)	
**Ovarian**			0.63		0.51
HGSOC	72 (71.3)	20 (76.9)		11 (84.6)	
Other	29 (28.7)	6 (23.1)		2 (15.4)	
**Stage**					
**Breast**			0.44		0.84
I-II	107 (44.2)	16 (53.3)		11 (40.7)	
III	135 (55.8)	14 (46.7)		16 (59.3)	
**Ovarian**			1		0.25
I-II	16 (16.5)	4 (15.4)		4 (30.8)	
III-IV	81 (83.5)	22 (84.6)		9 (69.2)	
**Chemotherapy**					
**Breast**			0.40		0.050
Antracyclines + Alkylating agents	221 (89.1)	30 (93.8)		20 (74.1)	
Alkylating agents only	21 (8.5)	1 (3.1)		6 (22.2)	
Antracyclines only	4 (1.6)	0 (0)		0 (0)	
Other	2 (0.8)	1 (3.1)		1 (3.7)	
**Ovarian**			0.67		0.29
Carboplatin	5 (5.1)	0 (0)		2 (15.4)	
Carbotaxol	92 (93.9)	26 (100)		11 (84.6)	
Other	1 (1)	0 (0)		0 (0)	

### Tumor characteristics and treatment

Among 304 breast cancer patients, 93 (30%) had triple-negative breast cancer and 22 (68%) of *gBRCA1* breast cancer patients developed triple-negative breast cancer. Among these breast cancer patients 221 were previously described [17], and we added 86 new patients (9 *gBRCA1*, 5 *gBRCA2* and 72 non-carriers). The large majority of the patients received doublet chemotherapy that included at least one DNA damaging agent: either platinum and taxane (94% of ovarian cancer patients) or cyclophosphamide and anthracyclines (88% of breast cancer patients; Table 1).

### Acute hematologic toxicities

Overall, 19/447 (4%) experienced a febrile neutropenia event after the first cycle of chemotherapy: 5/58 *gBRCA1* (8.6%; *p* = 0.16), 1/40 *gBRCA2* (2.5%) and 13/349 non-carriers (3.7%). The incidence of severe neutropenia (grade 4) after the first cycle was more frequent among *gBRCA1* (32.6%, *p* = 0.007). Most *gBRCA1* (58.3%; *p* = 0.011) needed secondary prophylaxis with G-CSF to complete their neoadjuvant or adjuvant chemotherapy, but this was not the case for *gBRCA2* carriers and non-carriers (Table 2). Overall, *gBRCA1* but not *gBRCA2* carriers were at higher risk of developing grade 3–4 neutropenia and requiring G-CSF to complete their adjuvant or neoadjuvant chemotherapy (Table 3).

**Table 2:** Incidence of acute hematological toxicities according to germline mutational status of *BRCA1/BRCA2* in the entire cohort

	Non-carriers	*BRCA1*	*p*	*BRCA2*	*p*
**Neutrophiles D8 (median ; min-max)**	1.8 (0–14.7)	1.2 (0–10.3)	0.067	1.9 (0.2–5.9)	0.33
**Grade 3–4**			0.034		0.55
No	214 (70.6)	23 (53.5)		27 (77.1)	
Yes	89 (29.4)	20 (46.5)		8 (22.9)	
**Grade 4**			0.007		1
No	259 (85.5)	29 (67.4)		30 (85.7)	
Yes	44 (14.5)	14 (32.6)		5 (14.3)	
**G-CSF**			0.011		0.72
No	197 (61.8)	20 (41.7)		21 (58.3)	
Yes	122 (38.2)	28 (58.3)		15 (41.7)	
**Dose reduction**			0.20		0.48
No	246 (82.3)	30 (73.2)		30 (88.2)	
Yes	53 (17.7)	11 (26.8)		4 (11.8)	
**Febrile neutropenia**			0.16		1
No	336 (96.3)	53 (91.4)		39 (97.5)	
Yes	13 (3.7)	5 (8.6)		1 (2.5)	

**Table 3:** Risk for developing acute hematological toxicities according to germline mutational status of *BRCA1/BRCA2* in the entire cohort

	*BRCA1*		*BRCA2*	
	**OR (95% CI)**	***p***	**OR (95% CI)**	***p***
**Grade 3–4 neutropenia after 1st cycle of chemotherapy**	2.4 (1.2 ; 4.8)	0.01	0.7 (0.3 ; 1.6)	0.46
**Grade 4 neutropenia after 1st cycle of chemotherapy**	3.3 (1.6 ; 7)	0.001	1 (0.3 ; 2.6)	0.97
**Febrile neutropenia after 1st cycle of chemotherapy**	2.5 (0.8 ; 7.1)	0.10	0.7 (0 ; 3.5)	0.69
**Dose reduction of chemotherapy**	1.4 (0.6 ; 3.3)	0.44	0.5 (0.1 ; 1.5)	0.23
**G-CSF use during chemotherapy**	2.5 (1.4 ; 4.8)	0.004	1.2 (0.6 ; 2.4)	0.66

Furthermore, we observed that *gBRCA1* breast cancer patients, but not ovarian cancer ones, were at risk for developing acute hematologic toxicities (**supplementary Tables **2 and 3).

#### Therapy-related myeloid neoplasms

After a median follow-up of 8 years in breast cancer cohort and 5 years in ovarian cancer cohort, we observed 2 cases of therapy-related myeloid neoplasms: one *gBRCA1* ovarian cancer patient who received chemotherapy and PARP inhibitor and one non-carrier breast cancer patient upon exposure to chemotherapy. The clinical and genomic characteristics of therapy-related myeloid neoplasms are described in Table 4.

**Table 4:** Clinical and genomic characteristics of patients who developed therapy-related myeloid neoplasms. t-MN : therapy-related myeloid neoplasms

	Type of cancer	g*BRCA* status	Age at diagnosis of tMN	Number of previous lines of chemotherapy	PARPi	Delay to tMN (years)	Type of tMN	Somatic mutations	Karyotype
**Patient #1**	breast	non-carrier	72	1	no	3	AML2	t(8;21) *RUNX1-RUNX1T1* transcript	complex
**Patient #2**	ovarian	g*BRCA1*	69	6	yes	15	MDS	*TP53*	complex

## Discussion

In the current study, we report that g*BRCA1* carriers but not g*BRCA2* carriers are at high risk of developing grade 3–4 neutropenia and are more likely to need secondary prophylaxis with G-CSF to complete their neoadjuvant or adjuvant chemotherapy. Increased risk of developing acute hematologic toxicities was observed only in breast cancer patients.

Our study was based on a biological hypothesis: we questioned whether the haploinsufficiency of the non-cancerous cells (here neutrophils) of women carrying g*BRCA* would induce greater sensitivity to DNA damage [13, 14]. This might be manifested by an increased incidence of acute hematologic toxicities upon exposure to myelosuppressive treatments such as chemotherapy.

Febrile neutropenia is a life-threatening consequence of chemotherapy. It increases mortality risk by 15% compared to patients with the same treatment. In our cohort of breast and ovarian cancer, 4.3% (19/447) of the patients developed febrile neutropenia, but this frequency increased to 8.6% among g*BRCA1* carriers. Furthermore, we found that the majority (58.3%) of g*BRCA1* carriers needed secondary prophylaxis with G-CSF to complete their adjuvant or neoadjuvant chemotherapy, while this was less the case for g*BRCA2* carriers and non-carriers.

Recently, post-hoc subgroup analyses of several randomized trials addressed whether g*BRCA* carriers are at higher risk for acute hematologic toxicities. The largest study in breast cancer patients was reported from the German Breast Group, which pooled several randomized trials’ data. They included only patients with triple-negative breast cancer (n = 1’171), of whom 210 were g*BRCA*. They found that g*BRCA* carriers (84% were in fact g*BRCA1*) were at high risk for developing acute hematologic toxicities if they received taxanes [18]. One limitation of the GBG analyses is that almost 40% of the patients received primary G-CSF prophylaxis. Another post-hoc subgroup analysis in the randomized phase III trial BROCADE3 investigating the combination of PARP inhibitor veliparib with carboplatin/paclitaxel in advanced breast cancer stage among g*BRCA* carriers found that anemia and thrombocytopenia were more frequent among g*BRCA1* than g*BRCA2* carriers [26].

For ovarian cancer, two post-hoc subgroup analyses from randomized phase III trials evaluating platinum/taxane doublet therapy combined with PARP inhibitor veliparib were recently published [19, 20]. Both studies did not show any increase of hematologic toxicities among g*BRCA* carriers, compared to non-carriers in the chemotherapy arm or chemotherapy and PARPi combination arm [20]. These observations are consistent with our subgroup analysis ovarian vs. breast cancer, where only g*BRCA1* breast cancer carriers were at risk for developing acute hematologic toxicities. This finding is intriguing since ovarian cancer patients receive platinum as frontline chemotherapy. A plausible explanation is that most breast cancer patients received 2 DNA damage agents: alkylating agent cyclophosphamide that induces DNA inter-strand crosslinks lesions, similarly to platinum [27], and a topoisomerase II inhibitor anthracycline.

Besides acute hematologic toxicity such as febrile neutropenia, it will be important to investigate whether g*BRCA* carriers are at higher risk for developing t-MN such as myelodysplastic syndrome and acute myeloid leukemia. tMN are rare but life-threatening events. With the recent approval of PARP inhibitors as frontline maintenance therapy in ovarian and breast cancer patients with g*BRCA* variants [28–30], this question becomes particularly important in these curable cancers [31, 32]. In breast cancer patients, the risk of t-MN is highest in older women who received anthracyclines-based chemotherapy [33, 34]. Few reports suggested an increased incidence of t-MN among g*BRCA* carriers [35, 36]. However, these reports were not case-control studies and were limited in their follow-up. Few case reports from the first trials investigating PARP inhibitors in ovarian cacer patients suggested that t-MN could be a delayed adverse event [22]. In the SOLO2 trial that included only ovarian cancer patients carrying g*BRCA*, t-MN occurred in 8% of patients receiving olaparib and 4% of those receiving placebo [21], raising concerns on the safety of long-term use of PARPi in g*BRCA* carriers. Consistently, a retrospective case-control analysis of ovarian cancer patients enrolled in the ARIEL2 and ARIEL3 trials suggested an increased incidence of tMN among patients carrying pathogenic variants in genes involved in homologous recombination pathway (*BRCA1*, *BRCA2, RAD51C* and *RAD51D*) [24]. A recent systematic review and safety meta-analysis of 28 randomized controlled trials comparing PARP inhibitors to placebo reported an increased risk (two to three-fold) for tMN in cancer patients treated with PARP inhibitors. Most cases (85%) were reported in ovarian cancer trials, likely due to the longest follow-up in completed trials in this disease (2–6 years). However, this meta-analysis did not find a significantly increased risk of t-MN among g*BRCA* carriers.

Genomic studies brought new insights into the pathogenesis of t-MN. They support a model where cytotoxic therapy does not directly induce tMN. Rather, clonal hematopoiesis precedes cancer therapy [37–39]. DNA damaging agents such as platinum and topo-isomerase II inhibitors preferentially select clones enriched in mutated DNA damage response genes (*TP53, PPM1D* and *CHEK2*)[38] that expand and transform into t-MN, and this holds true for PARP inhibitors [39]. Indeed, it was shown that clonal hematopoiesis preceded t-MN and expanded in ovarian cancer patients treated with PARP inhibitors [24]. These t-MN harbor, similarly to those arising with chemotherapy, pathogenic variants in DNA damage response genes and are characterized by complex karyotypes [39].

Our study has several limitations. It is a retrospective monocentric study with a limited number of patients diagnosed over 15 years. The hematological data collected on days 7–14 do not always reflect toxicity. All the patients included met the criteria for germline genetic screening and this population is, therefore, not representative of all patients with breast or ovarian cancer. Additionally, genetic testing techniques have recently changed from Sanger sequencing to next-generation sequencing. Patient records included in our study were not all located in the same establishment. Chemotherapy regimens were not homogeneous, as we included both breast and ovarian cancer patients. This variability in the chemotherapy regimen is an important bias for the ovarian occurrence of hematologic toxicity because it reduces the dose intensity.

Nevertheless, our observations were consistent with recent post-hoc subgroup analyses from randomized trials in breast and ovarian cancer patients. Further investigation of tMN occurrence is warranted with the recent approval of PARPi in frontline maintenance therapy in curable cancers. Biobanks of prospective and longitudinal samples collected during PARPi trials are unique resources to investigate whether exposure to PARPi shapes clonal hematopoiesis toward t-MN [24, 40], and whether this effect may be more frequent among g*BRCA1* or g*BRCA2* carriers.

## Electronic supplementary material

Below is the link to the electronic supplementary material.

Supplementary Material 1

Supplementary Material 2

## Data Availability

Data might be made available upon request and approval by Geneva ethics committee.
