# Supplementary material for: Hematologic toxicities of chemotherapy in breast and ovarian cancer patients carrying BRCA1/BRCA2 germline pathogenic variants. A single center experience and review of the literature
Source: Fam Cancer. 2023 Apr 29;22(3):283–9. doi: 10.1007/s10689-023-00331-6 (PMC10276105; doi:10.1007/s10689-023-00331-6)
Supplement: Supplementary file 1 — Supplementary Material 1 [file 10689_2023_331_MOESM1_ESM.pptx]

## Slide 1
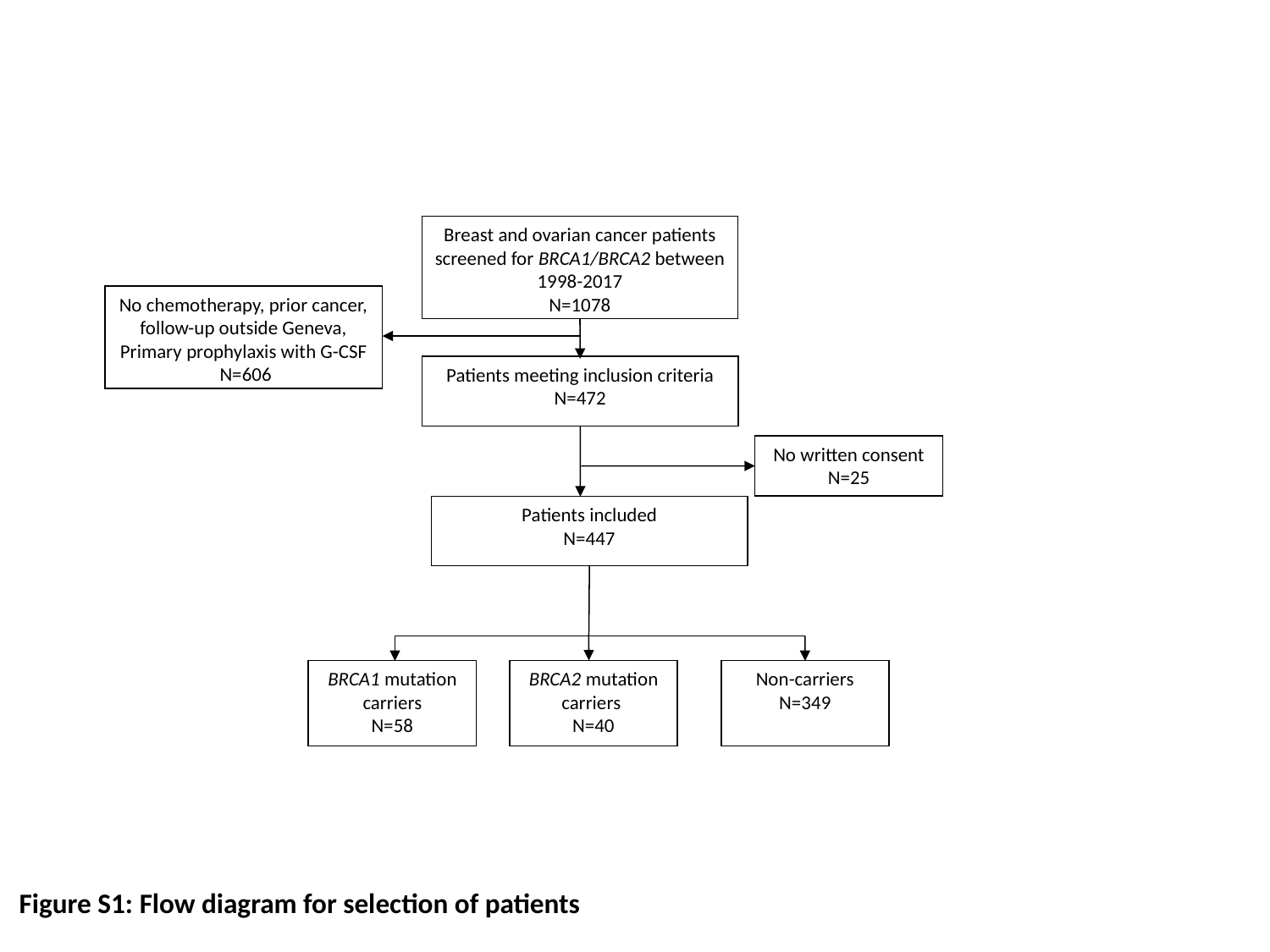

Breast and ovarian cancer patients screened for BRCA1/BRCA2 between 1998-2017
N=1078
No chemotherapy, prior cancer, follow-up outside Geneva,
Primary prophylaxis with G-CSF
 N=606
Patients meeting inclusion criteria
N=472
No written consent
N=25
Patients included
N=447
BRCA1 mutation carriers
N=58
BRCA2 mutation carriers
N=40
Non-carriers
N=349
Figure S1: Flow diagram for selection of patients
