# Supplementary material for: Hematologic toxicities of chemotherapy in breast and ovarian cancer patients carrying BRCA1/BRCA2 germline pathogenic variants. A single center experience and review of the literature
Source: Fam Cancer. 2023 Apr 29;22(3):283–9. doi: 10.1007/s10689-023-00331-6 (PMC10276105; doi:10.1007/s10689-023-00331-6)
Supplement: Supplementary file 2 — Supplementary Material 2 [file 10689_2023_331_MOESM2_ESM.docx]

|  | **Overall** | | **Breast** | | **Ovarian** | |
| --- | --- | --- | --- | --- | --- | --- |
|  | **n** | **%** | **n** | **%** | **n** | **%** |
| **Age** | 1 | 0.2 | 0 | 0 | 0 | 0 |
| **Histology** | 3 | 0.7 | 3 | 1 | 0 | 0 |
| **Stage** | 12 | 2.7 | 8 | 2.6 | 4 | 2.9 |
| **Treatment** | 3 | 0.7 | 0 | 0 | 3 | 2.1 |
| **Neutrophiles post-treatment** | 66 | 14.8 | 38 | 12.4 | 28 | 20 |
| **Dose reduction** | 73 | 16.3 | 39 | 12.7 | 34 | 24.3 |
| **GCSF** | 44 | 9.8 | 25 | 8.1 | 19 | 13.6 |
| **Febrile neutropenia** | 0 | 0 | 0 | 0 | 0 | 0 |

**Supplementary Table 1: Missing data of the study variables**

|  | ***BRCA1*** |  | ***BRCA2*** |  |
| --- | --- | --- | --- | --- |
| **Breast cancer** | **OR (95% CI)** | ***p*** | **OR (95% CI)** | ***p*** |
| **Grade 3–4 neutropenia after 1st cycle of chemotherapy** | 2.5 (1.1 ; 5.9) | 0.031 | 1 (0.4 ; 2.4) | 0.98 |
| **Grade 4 neutropenia after 1st cycle of chemotherapy** | 3.9 (1.6 ; 9.3) | 0.0021 | 1.3 (0.4 ; 3.5) | 0.62 |
| **Febrile neutropenia after 1st cycle of chemotherapy** | 5.9 (1.4 ; 22.1) | 0.0089 | 1.6 (0.1 ; 9.7) | 0.69 |
| **Dose reduction of chemotherapy** | 2.5 (0.5 ; 8.5) | 0.19 | 1.6 (0.2 ; 6.3) | 0.57 |
| **G-CSF use during chemotherapy** | 4.2 (1.8 ; 11) | 0.002 | 2 (0.9 ; 4.6) | 0.11 |

**Supplementary Table 2: Risk for developing acute hematological toxicities according to germline status of *BRCA1/BRCA2* in breast cancer patients.**

|  | ***BRCA1*** |  | ***BRCA2*** |  |
| --- | --- | --- | --- | --- |
| **Ovarian cancer** | **OR (95% CI)** | ***p*** | **OR (95% CI)** | ***p*** |
| **Grade 3–4 neutropenia after 1st cycle of chemotherapy** | 2.2 (0.7 ; 6.7) | 0.182 | NA | NA |
| **Grade 4 neutropenia after 1st cycle of chemotherapy** | 2.3 (0.4 ; 9.9) | 0.28 | NA | NA |
| **Febrile neutropenia after 1st cycle of chemotherapy** | 0.6 (0 ; 4) | 0.662 | NA | NA |
| **Dose reduction of chemotherapy** | 1.2 (0.4 ; 3.8) | 0.71 | 0.2 (0 ; 0.9) | 0.056 |
| **G-CSF use during chemotherapy** | 1.3 (0.5 ; 3.5) | 0.567 | 0.2 (0 ; 1) | 0.113 |

**Supplementary Table 3: Risk for developing acute hematological toxicities according to germline status of *BRCA1/BRCA2* in ovarian cancer patients.**
